# Phospholipid flippases attenuate LPS-induced TLR4 signaling by mediating endocytic retrieval of Toll-like receptor 4

**DOI:** 10.1007/s00018-016-2360-5

**Published:** 2016-09-14

**Authors:** Vincent A. van der Mark, Mohammed Ghiboub, Casper Marsman, Jing Zhao, Remco van Dijk, Johan K. Hiralall, Kam S. Ho-Mok, Zoë Castricum, Wouter J. de Jonge, Ronald P. J. Oude Elferink, Coen C. Paulusma

**Affiliations:** Tytgat Institute for Liver and Intestinal Research, Academic Medical Center, Meibergdreef 69-71, 1105 BK Amsterdam, The Netherlands

**Keywords:** P4-ATPase, Phospholipid flippase, ATP8B1, Endocytosis, Innate immunity, Macrophage

## Abstract

P4-ATPases are lipid flippases that catalyze the transport of phospholipids to create membrane phospholipid asymmetry and to initiate the biogenesis of transport vesicles. Here we show, for the first time, that lipid flippases are essential to dampen the inflammatory response and to mediate the endotoxin-induced endocytic retrieval of Toll-like receptor 4 (TLR4) in human macrophages. Depletion of CDC50A, the β-subunit that is crucial for the activity of multiple P4-ATPases, resulted in endotoxin-induced hypersecretion of proinflammatory cytokines, enhanced MAP kinase signaling and constitutive NF-κB activation. In addition, CDC50A-depleted THP-1 macrophages displayed reduced tolerance to endotoxin. Moreover, endotoxin-induced internalization of TLR4 was strongly reduced and coincided with impaired endosomal MyD88-independent signaling. The phenotype of CDC50A-depleted cells was also induced by separate knockdown of two P4-ATPases, namely ATP8B1 and ATP11A. We conclude that lipid flippases are novel elements of the innate immune response that are essential to attenuate the inflammatory response, possibly by mediating endotoxin-induced internalization of TLR4.

**Electronic supplementary material:**

The online version of this article (doi:10.1007/s00018-016-2360-5) contains supplementary material, which is available to authorized users.

## Introduction

Toll-like receptor 4 (TLR4) is expressed on myeloid-derived cells, including macrophages and dendritic cells, and on some epithelial and endothelial cells and is an essential component of the innate immune response [1]. Activation of TLR4 involves multiple co-receptors, including lipopolysaccharide-binding protein, myeloid differentiation protein 2 and CD14. These co-receptors mediate ligation and transfer of lipopolysaccharides (LPS), an inflammatory mediator of the cell wall of Gram-negative bacteria, to TLR4 [2]. This ligation induces dimerization of TLR4 and activates a MyD88-dependent early response signaling pathway that results in the production of pro-inflammatory cytokines such as TNFα, IL-1β and IL-6 [3]. Consequently, TLR4 is endocytosed, which induces MyD88-independent signaling from the TLR4-containing endosomal recycling compartment [4–6], inflicting a type I interferon response that is critical to counter an ongoing infection [7]. Importantly, TLR4 internalization reduces signaling from the plasma membrane and dampens the inflammatory reaction. Failure to curtail the TLR4-mediated immune response can lead to pervasive tissue injury and may give rise to immunopathology such as sepsis, autoimmune diseases, metabolic diseases, neurodegeneration and chronic inflammation [1, 8]. Although the molecular mechanisms underlying the endocytic retrieval of TLR4 are poorly understood, it is a dynamin-driven process that strongly depends on CD14 [5, 9].

P4-ATPases are a family of phospholipid flippases, i.e., integral membrane proteins that translocate phospholipids from the exoplasmic leaflet to the cytoplasmic leaflet of biological membranes [10–12]. Emerging evidence in *S. cerevisiae*, *C. elegans*, *A. thaliana* and mammalian cells indicate important functions for members of the P4-ATPase protein family in the biogenesis of intracellular transport vesicles in the biosynthetic and endocytic pathways [10, 13]. For instance, yeast cells deficient for the P4-ATPase Drs2p have a defect in the biogenesis of clathrin-coated vesicles at the *trans*-Golgi network (TGN), while combined deficiency of different P4-ATPases leads to defects in fluid-phase endocytosis and intracellular protein transport [14–16]. Similarly, P4-ATPases in *C. elegans* have been implicated in receptor-mediated and fluid-phase endocytosis and in endocytic sorting and recycling [17, 18], while in the plant *A. thaliana*, P4-ATPases are involved in generation of a specific class of TGN-derived secretory vesicles important for root development [19]. In mammalian cells P4-ATPases probably also fulfill important roles in vesicle biogenesis, but evidence for this is much more scarce. For instance, ATP11B is thought to confer resistance to cis-platinum in ovarian cancer via the biogenesis of cisplatin-containing vesicles at the TGN [20]. ATP8B1 mediates the apical targeting of the apical sodium-dependent bile acid transporter SLC10A2/ASBT, either directly from the TGN or via recycling from a subapical vesicle pool in intestinal Caco-2 cells [21]. Recently, ATP8A1 and ATP8A2 were shown to be essential for membrane fission of recycling endosomes [22].

Thus far, deficiency of two P4-ATPases were shown to cause severe hereditary disease. Mutations in *ATP8B1* cause progressive familial intrahepatic cholestasis type 1, a severe liver disease characterized by impaired bile formation [23]. Mutations in *ATP8A2* are associated with a severe neurological disorder described as cerebellar axatia, mental retardation and dysequilibrium syndrome (CAMRQ) [24].

Most P4-ATPases function as a heterodimer with a member of the CDC50 protein family, in which the P4 ATPase is the α-subunit and CDC50 the β-subunit [11, 25]. The human genome encodes fourteen P4-ATPases and three CDC50 proteins, eleven of which form a heterodimer with CDC50A. CDC50A is a ~50-kDa complex-glycosylated transmembrane protein [26], and its interaction with individual P4-ATPases is essential for endoplasmic reticulum exit and activity of the heterodimer [27–30].

Here we have investigated the hypothesis that lipid flippases of the P4-ATPase family are important mediators of TLR4-mediated signaling. We have analyzed the inflammatory reaction of CDC50A-depleted THP-1 and primary human monocyte-derived macrophages upon LPS challenge. Our data point to important functions for multiple P4-ATPases in attenuating TLR4-mediated signaling.

## Materials and methods

### Cell culture and lentiviral transduction

The human monocytic leukemia cell line THP-1 was cultured in RPMI 1640 medium (Gibco) supplemented with 10 % fetal bovine serum (FBS) (Lonza), 2 mM l-glutamine (Lonza), 100 U/ml penicillin (Lonza), and 100 U/ml streptomycin (Lonza) at 37 °C in a 5 % CO_2_ humidified atmosphere. Knockdown cell lines for CDC50A and P4 ATPases were generated by lentiviral transduction of undifferentiated THP-1 cells [31]. Briefly, 0.5 × 10^6^ cells were incubated with virus-containing supernatants/RPMI 1640 (1:1) supplemented with 10 µg/ml diethylaminoethyldextran for 4 h. Two days post-transduction, cells were selected with 2 µg/ml puromycin. Validated short-hairpin RNA (shRNA) vectors to *CDC50A* [TRCN0000159317 (4) and TRCN0000160267 (1)], *ATP8B1* (TRCN0000050127) and *ATP11A* (TRCN0000051887) were obtained through the MISSION shRNA library (Sigma-Aldrich). SHC002 was included as a control.

Lentiviral constructs to haemagglutinin antigen (HA)-tagged *CDC50A* (*HA*-*CDC50A*) and enhanced green fluorescent protein (eGFP)-tagged *ATP8B1* (*ATP8B1*-*eGFP*) were described previously [27]. A Rab11-eGFP plasmid [32] was AgeI/XbaI-digested and subcloned into a second generation lentiviral transfer vector [31]. *ATP11A* cDNA was obtained from the PlasmID Repository/DF/HCC DNA Resource Core (http://plasmid.med.harvard.edu). A FLAG-tag was introduced on the 3′ end by PCR using forward oligo 5′-cattagctacgaccggtatggactgcagcctcgtgcggacg-3′ and reverse oligo 5′-ggctggtctagactaCTTGTCATCGTCGTCCTTGTAGTCgaaactcaggctgctggaag-3′, in which the FLAG sequence is capitalized. All experiments were performed on THP-1 cells differentiated to macrophages with 100 nM phorbol-12-myristate-13-acetate (PMA) (Sigma-Aldrich) for 3 days, after which they were rested for 2–3 days in PMA-free culture medium [33]. Differentiated THP-1 cells were challenged with LPS from *Escherichia coli* 0111:B4 (Sigma-Aldrich) at 37 °C, 5 % CO_2_ for indicated time-points and at indicated concentrations.

### Isolation, maturation, polarization and siRNA transfection of monocyte-derived macrophages

Peripheral blood mononuclear cells (PBMCs) were obtained from whole blood of healthy donors by density gradient centrifugation using Ficoll (Invitrogen). Briefly, 13 ml Ficoll was added below 30 ml PBS-diluted blood and centrifuged at 2000 rpm for 20 min at room temperature, with acceleration 3 and no break. The interphase was recovered using a Pasteur pipet into a new 50 ml tube containing 10 ml PBS, followed by twice washing with PBS. 5 × 10^6^ of PBMCs (containing ~0.5 × 10^6^ monocytes) were incubated in 12-well plates in 1 ml Isocove’s Modified Dulbecco’s Medium (Lonza) supplemented with 10 % fetal bovine serum (FBS) (Lonza), 2 mM l-glutamine (Lonza), 100 U/ml penicillin (Lonza) and 100 U/ml streptomycin (Lonza) for 90 min at 37 °C, 5 % CO_2_. After 2 h, medium was aspirated and the cells were washed several times with sterilized warm PBS till removing all floating cells. Monocytes were matured using 72 h treatment with 20 ng/ml of macrophage colony-stimulating factor (M-CSF). The cells were then washed with PBS and stimulated with 100 ng/ml IFNγ or 40 ng/ml IL-4 for 3 days to generate M1 or M2 macrophages, respectively. Medium was added to maturated monocytes (M0 macrophages) for 3 days to keep it as M0 subset. M1 macrophages were transfected using DharmaFECT™ transfection reagents (Dharmacon) according to the manufacturer’s instructions with siGENOME human TMEM30A smartpool siRNAs to deplete CDC50A and non-targeting siRNAs as a control. Cells were analyzed at 72 h post-transfection.

### Enzyme linked immuno sorbent assay

ELISA (R&D Systems) for human TNFα, IL1β and IL6 were performed according to the manufacturer’s instructions.

### Quantitative RT-PCR

Total RNA was isolated from differentiated cells using TriPURE reagent (Invitrogen). cDNA was synthesized from 2 μg of total RNA with random hexamers and oligo dT 12–18 primer and Superscript III RT (Invitrogen). Real-time PCR measurements were performed on a Lightcycler 480 (Roche) with Fast Start DNA MasterPlus SYBR Green I kit (Roche). Expression levels in THP-1 cells and human monocyte-derived M1 macrophages were calculated with the LinregPCR software [34] and were normalized to the geometric means of the three most stable reference genes (RPLP0, ACTB, GAPDH) as determined by Genorm analysis [35]. Expression levels in primary mouse cells were calculated similarly and were normalized to Rplp0. Primer sequences are depicted in supplemental Table 1.

### Isolation of nuclear extracts

Cells were washed with ice-cold PBS, scraped into solution and pelleted by centrifugation (400×*g*, 10 min, 4 °C). The cell pellet was washed in 5 packed cell volumes (PCV) resuspension buffer (10 mM HEPES pH 7.9, 1.5 mM MgCl_2_, 10 mM KCl, 0.5 mM DTT) supplemented with 0.5 mM phenylmethanesulfonylfluoride, phosSTOP phosphatase and protease inhibitor cocktails (Roche) and centrifuged (400×*g*, 10 min, 4 °C). The cell pellet was incubated for 10 min on ice in 2 PCV resuspension buffer and homogenized with a Dounce homogenizer (tight pestle, 60 strokes). Cell lysis was confirmed via microscopic analysis and non-broken cells were pelleted by centrifugation (200×*g*, 2 min, 4 °C). The resulting supernatant was pelleted again by centrifugation (425×*g*, 10 min, 4 °C). The supernatant containing the cytosolic fraction was stored on ice and the nuclei-containing pellet was resuspendend in ultracentrifuge resuspension buffer (20 mM HEPES pH 7.9, 1.5 mM MgCl_2_, 0.42 M NaCl, 0.5 mM DTT, 0.2 mM EDTA, 25 % v/v glycerol, 0.5 mM phenylmethanesulfonylfluoride, phosSTOP phosphatase and protease inhibitor cocktails) and incubated while rotating for 30 min at 4 °C. The nuclear and cytosolic fractions were each separately subjected to ultracentrifugation in an Optima L-90K ultracentrifuge (Beckton Dickenson, Ti70 rotor, 32,000×*g*, 30 min, 4 °C). Cleared supernatants were stored at −80 °C until use.

### SDS-PAGE and western blotting

Cells were lysed in RIPA buffer (50 mM Tris–HCl, pH 8.0, 150 mM NaCl, 1 % NP-40, 0.5 % Na-deoxycholate, 0.1 % SDS) containing protease inhibitor cocktail (Roche) and/or PhosSTOP phosphatase inhibitor cocktail (Roche). Proteins were separated on a polyacrylamide gel (6–12 % depending on the size of the protein of interest) and were transferred to Immobilon-P PVDF membranes (Millipore) by semi-dry blotting using 10 mM CAPS, pH 10.5/15 % methanol buffer. Membranes were blocked for 1 h at RT in block buffer [PBS/5 % low-fat milk (Nutricia Profitar-plus)] and incubated for 1 h at RT in block buffer with rabbit polyclonal antibodies to TLR4 (H-80, Santa Cruz), CDC50A [26], ATP8B1 [36], histone H3 [(di methyl K79) antibody—ChIP Grade (Abcam)], ATP1A1 [37], phospho-SAPK/JNK (Thr183/Tyr185), SAPK/JNK, phospho-p44/42 MAPK (Erk1/2) (Thr202/Tyr204), p44/42 MAPK (Erk1/2), mouse monoclonal to phospho-NF-kB p65 (Ser536) (7F1) (all from Cell Signaling), NF-κB p65 (F-6) (Santa Cruz), GAPDH (MAB374, Millipore), and β-actin (clone AC-15, Sigma Aldrich). Immune complexes were visualized with peroxidase-conjugated goat-anti-rabbit or mouse IgGs (Bio-Rad), developed with homemade enhanced chemiluminescence reagents (100 mM Tris–HCl, pH 8.5, 1.25 mM luminol, 0.2 mM *p*-coumarin and freshly added 3 mM H_2_O_2_) and detected using an ImageQuant™ LAS 4000 (GE Healthcare). Densitometric analyses of band intensities were performed using ImageJ 1.49.

### Indirect immunofluorescence

Differentiated THP-1 cells (knockdowns, HA-CDC50A/Rab11eGFP-, ATP8B1eGFP- and ATP11A-Flag over-expressing) were grown on glass coverslips and fixed in 2 % paraformaldehyde (PFA) for 20 min at RT. PFA fixed cells were permeabilized on PBS/0.1 % triton X-100 (PBS-Tx) and incubated with rat monoclonal anti-HA (clone 3F10; Roche) to detect CDC50A, mouse monoclonal anti-Flag (M2, Sigma) to detect ATP11A or rabbit polyclonal anti-TLR4 (H-80, Santa Cruz). Cells were extensively washed in between antibody incubations in PBS-Tx. Immunoreactivity was visualized with goat-anti-rat Texas Red, goat-anti-mouse Alexa 488 or goat-ant-rabbit Alexa 594 (Molecular Probes). Sections were mounted in Vectashield/DAPI (Vector Laboratories) and were imaged on a SP8-X-SMD confocal microscope (Leica) or on a DMi8 microscope (Leica).

### Measuring TLR4/CD14/CD11B surface expression by flow cytometry

PMA-differentiated THP-1 cells (2 × 10^6^/well, 6-well plate) were washed with ice-cold PBS and were incubated in ice-cold PBS containing 5 mM ethylenediaminetetraacetic acid (PBS/EDTA) for 10 min on ice. Cells were removed using a cell scraper (Corning) and were transferred to eppendorf tubes. Cells were centrifuged for 5 min at 240×*g* and supernatant was removed after which the cell pellet was incubated with mouse IgG2a phycoerythrin (PE)-conjugated anti-human CD284 (TLR4) antibody (clone HTA125, BioLegend, San Diego, USA), mouse IgG2b PE-conjugated anti-human CD14 (clone MϕP9, BD Biosciences) or mouse IgG1 Alexa Fluor 488-conjugated anti-human CD11B (clone ICRF44, BD Biosciences) in PBS for 45 min at 4 °C in the dark with constant agitation. Subsequently cells were washed with ice-cold PBS, were resuspended in PBS and immediately analyzed by flow cytometry using a BD LSRFortessa™ cell analyzer (BD Biosciences). To analyze LPS-induced TLR4/CD14 internalization, differentiated THP-1 cells and human monocyte-derived M1 macrophages were challenged with LPS (concentration indicated in figure legend) at 37 °C, 5 % CO_2_ for indicated time-points. Cells were analyzed as described above.

### Statistics

Data are displayed as mean ± standard deviation (sd) or standard error of the mean (sem). Statistical significance was determined by performing Student’s *t* test or one-way ANOVA with Bonferroni’s correction for multiple testing as indicated in the legend of the figures.

## Results

### CDC50A is required to dampen the inflammatory reaction triggered by LPS in human macrophages

**Fig. 1:**
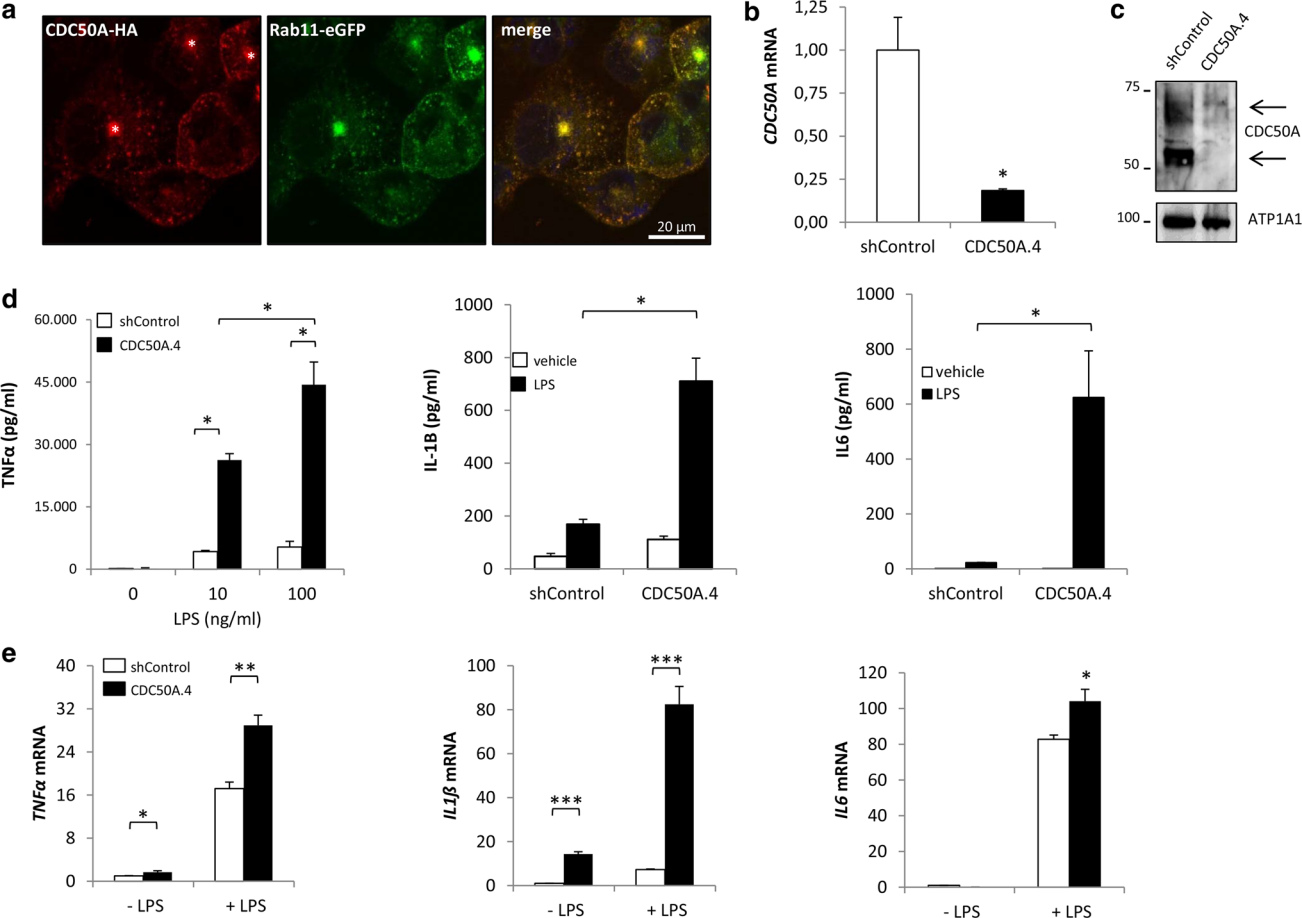
CDC50A depletion leads to endotoxin-induced hypersecretion of inflammatory cytokines in THP-1 macrophages. **a** Confocal microscopical detection of HA-tagged CDC50A and GFP-tagged Rab11 in THP-1 macrophages. CDC50-HA co-stained with Rab11eGFP-positive (recycling) endosomes and the endocytic recycling compartment (indicated by an *asterisk* in merge). Nuclear DAPI staining in *blue*. **b** *CDC50A* mRNA levels in shControl and CDC50A-depleted THP-1 macrophages. Data shown are mean ± standard deviation of triplicate wells. mRNA expression in shControl cells was set as 1. Statistical significance was tested by a Student’s *t* test, *p < 0.002. **c** Immunoblot analysis of CDC50A protein levels in membrane isolates of shControl and CDC50A-depleted THP-1 macrophages. *Arrows* indicate the different glycosylation states of CDC50A. ATP1A1 is included as a loading control. **d** TNFα, IL1-β and IL6 secretion in shControl and CDC50A-depleted THP-1 macrophages 4 h after 100 ng/ml LPS administration. Data shown are mean ± standard deviation of quadruple wells. Statistical significance was tested by one-way ANOVA with Bonferroni’s correction for multiple testing, *p < 0.005. **e** *TNFα, IL*-*1β* and *IL6* mRNA expression levels in shControl and CDC50A-depleted THP-1 macrophages 3 h post-LPS administration. Data shown are mean ± standard deviation of triplicate wells. mRNA expression in shControl cells without LPS was set as 1. Statistical significance was tested by a Student’s *t* test, *p < 0.05, **p < 0.001, ***p < 0.0001. Data shown in this figure are representative of three to six independent experiments with similar results

To investigate the role of CDC50A-interacting P4-ATPases in the innate immune response in macrophages, we analyzed the inflammatory response to LPS challenge in human THP-1 and primary human monocyte-derived macrophages (MDMs) that were depleted from CDC50A. As we could not localize endogenous CDC50A, we assessed the localization of ectopically expressed HA-tagged CDC50A in differentiated THP-1 macrophages (Fig. 1a). HA-CDC50A localized to intracellular vesicles and almost complete costained with Rab11, a marker for recycling endosomes and the endocytic recycling compartment; in addition, weak plasma membrane staining was detected. Since CDC50A is only released from the endoplasmic reticulum in association with a P4-ATPase [28], this localization most likely represents a functional CDC50A-P4-ATPase association. Stable CDC50A-depleted THP-1 cells (further referred to as CDC50A.4 cells) were generated by constitutive expression of short hairpin RNA sequences targeting *CDC50A*. *CDC50A* mRNA expression was reduced by >80 % in CDC50A.4 cells compared to shControl cells (Fig. 1b), which coincided with strongly reduced CDC50A protein levels (Fig. 1c). CDC50A depletion did not interfere with cell growth, morphology or differentiation, the latter evidenced by normal CD11B surface expression (supplementary figure 1A). Differentiated THP-1 cells were incubated for 4 h with LPS and TNFα, IL-1β and IL6 secretion was quantified by ELISA. Compared to shControl cells, CDC50A.4 macrophages showed a strongly enhanced output (>6 fold) of TNFα, IL-1β and IL6 upon LPS challenge (Fig. 1d). This phenotype was reproduced in CDC50A-depleted THP-1 cells generated by a different shRNA sequence (supplementary figure 1B). The enhanced secretion of these cytokines was mirrored by their mRNA expression (Fig. 1e).

**Fig. 2:**
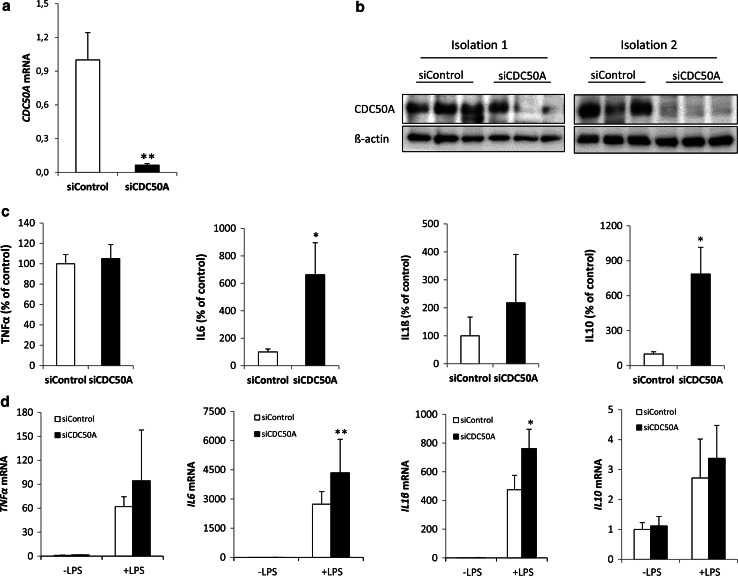
CDC50A depletion leads to endotoxin-induced hypersecretion of inflammatory cytokines in primary human monocyte-derived macrophages. **a** *CDC50A* mRNA levels in primary human monocyte-derived macrophages (MDM) transfected with scrambled siRNAs (siControl) and siRNAs directed to CDC50A (siCDC50A). RNA was isolated 72-h post-transfection. Data shown are mean ± standard deviation; *n* = 8 of two different isolations. Statistical significance was tested by a Student’s *t* test, **p < 0.0005. **b** Immunoblot analysis of CDC50A protein levels in total lysates of two independent isolations of MDMs. β-Actin is included as a loading control. **c** TNFα, IL6, IL1-β and IL10 secretion in siControl and siCDC50A MDMs (72 h post-transfection) that were stimulated for 4 h with 100 ng/ml LPS. Data shown are mean ± SEM of *n* = 10 of four different isolations. Statistical significance was tested by a Student’s *t* test, *p < 0.05 (**d**) *TNFα, IL6, IL*-*1β* and *IL10* mRNA expression levels in siControl and siCDC50A MDMs (72 h post-transfection) 3 h post-LPS (100 ng/ml) administration. Data shown are mean ± standard deviation (*n* = 8, 2 different isolations). mRNA expression in siControl cells without LPS was set as 1. Statistical significance was tested one-way ANOVA with Bonferroni’s correction for multiple testing, *p < 0.01; **p < 0.005

To verify the physiological relevance of the hyper-inflammatory phenotype observed in CDC50A-depleted THP-1 cells, we performed the same analyses in primary human MDMs in which CDC50A was transiently depleted by CDC50A-directed siRNA transfection. Analyses were performed in M1 polarized MDMs, M1 polarization evidenced by high expression of M1-specific marker *CD80* and absence of M2-specific marker *CD200R* (supplemental figure 1C) [38]. 72 Hour post-transfection MDMs were challenged for 4 h with LPS and the inflammatory response was assessed (Fig. 2). CDC50A mRNA expression and protein levels were strongly reduced 72 h post-transfection (Fig. 2a, b). In contrast to CDC50A-depleted THP-1 macrophages, CDC50A-depleted M1 macrophages displayed no elevated excretion of TNFα; However, IL6 and IL10 excretion was ~6 fold increased by these cells, while IL1β tended to be increased (Fig. 2c). Also at the mRNA level, expression of most of mentioned cytokines was slightly elevated (Fig. 2d). Collectively these data suggest that CDC50A-associated P4-ATPases negatively regulate TLR4-mediated signaling in human macrophages.

### CDC50A attenuates TLR4-mediated signaling in human macrophages

**Fig. 3:**
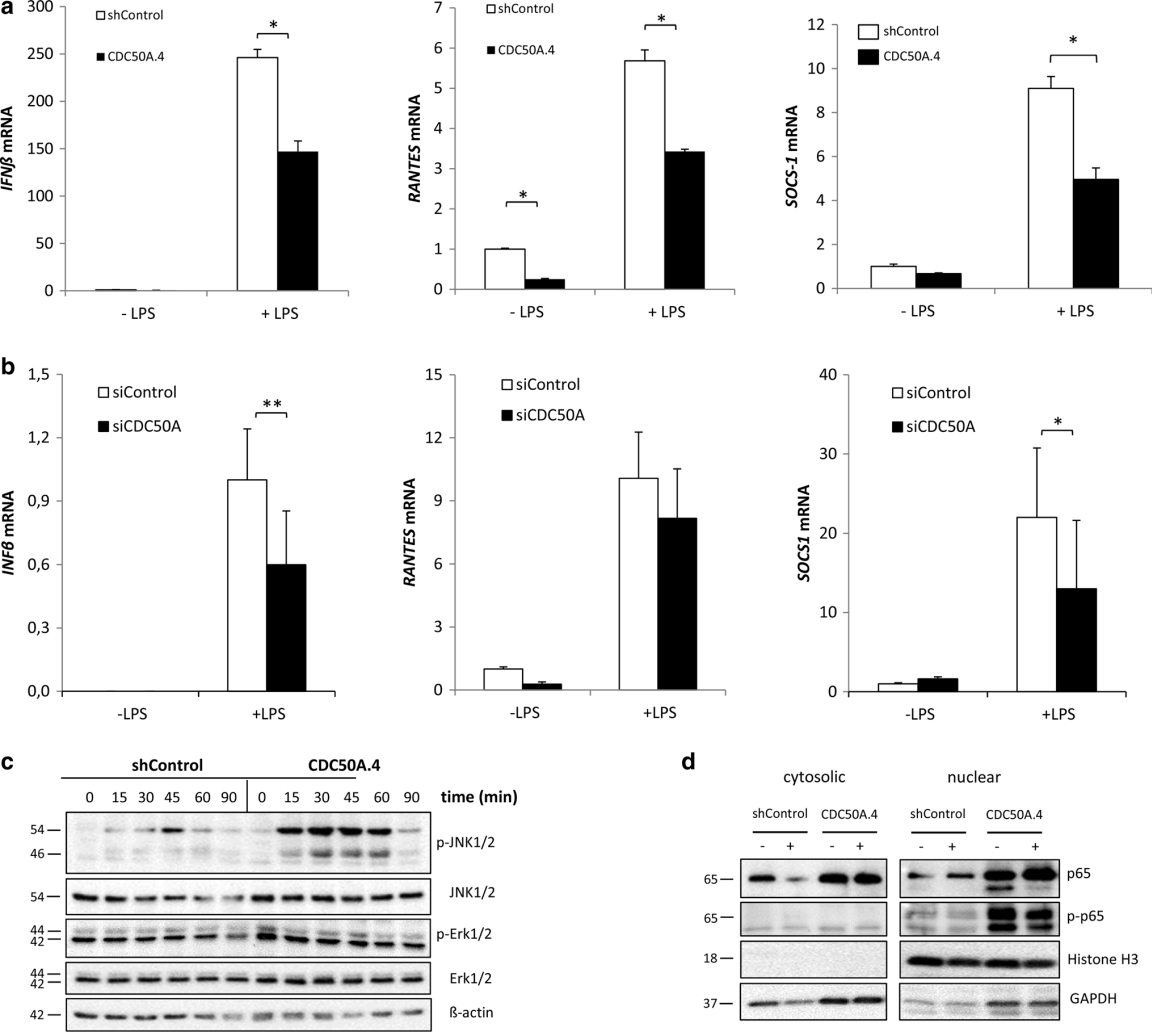
CDC50A depletion in human macrophages leads to an impaired interferon response and sustained TLR4-mediated signaling. **a** *IFNβ*, *RANTES* and *SOCS1* mRNA levels in shControl and CDC50A-depleted THP-1 cells 3 h post-LPS administration. Data shown are mean ± standard deviation of triplicate wells. mRNA expression in shControl cells without LPS was set as 1. Statistical significance was tested by a Student’s *t* test, *p < 0.0005. Data shown are representative of three to six independent experiments with similar results. **b** *IFNβ*, *RANTES* and *SOCS1* mRNA levels in siControl and siCDC50A MDMs (72 h post-transfection) 3 h post-LPS (100 ng/ml) administration. Data shown are mean ± standard deviation (*n* = 7, 2 different isolations). mRNA expression in siControl cells without LPS was set as 1. Statistical significance was tested one-way ANOVA with Bonferroni’s correction for multiple testing, *p < 0.05; **p < 0.005. **c** Immunoblot analysis of activated JNK1/2 and Erk1/2 in shControl and CDC50A-depleted THP-1 macrophages. Cells were incubated with 100 ng/ml LPS for the time points indicated. β-Actin is included as a loading control. **d** Immunoblot analysis of cytosolic and nuclear total and activated NF-κB p65 in shControl and CDC50A-depleted THP-1 macrophages. Cells were incubated with 10 ng/ml LPS for 2 h. GAPDH and histone H3 are included as cytosolic and nuclear loading controls, respectively

TLR4 activation by LPS leads to induction of the interferon signaling pathway from TLR4-containing endosomes [5, 6]. This late response is characterized by the induction of interferon β (*IFN*β) and *RANTES/CCL5*, which we analyzed in CDC50A-depleted THP-1 and primary MDM-derived macrophages (Fig. 3). In both control and CDC50A-depleted macrophages LPS challenge induced *IFNβ* and *RANTES*, however, the induction was significantly less in CDC50A-depleted THP-1 (Fig. 3a) and MDMs (Fig. 3b). Similarly, suppressor of cytokine signaling 1 (SOCS1), which is a negative regulator of TLR-mediated signaling to dampen the inflammatory reaction [39], was induced in LPS-challenged macrophages, however, this induction was significantly reduced in CDC50A-depleted THP-1 and MDMs, suggesting impaired negative feedback regulation of TLR4-mediated signaling (Fig. 3a, b). These data suggest that CDC50A plays an important role in the induction of the interferon response and in dampening the TLR4-mediated inflammatory reaction in macrophages. Since in both CDC50A-depleted THP-1 and primary human macrophages downstream signaling of TLR4 was affected, we further assessed the role of CDC50A in TLR4-mediated signaling in THP-1 macrophages. LPS-induced TLR4 signaling leads to nuclear translocation of the transcription factors AP-1 (via MAPK pathways) and NF-κB to promote transcription of pro-inflammatory cytokines. We thus analyzed activation (phosphorylation) of c-Jun terminal kinases (JNK) 1/2, extracellular signal-regulated kinases (ERK) 1/2 and NF-κB in LPS challenged cells (Fig. 3c). In CDC50A.4 macrophages JNK phosphorylation was strongly induced already 15 min after LPS challenge and was more sustained compared to shControl cells. Importantly, the levels of nuclear phosphorylated NF-κB were strongly increased in both LPS-stimulated and unstimulated CDC50A.4 macrophages (Fig. 3d). These observation provide additional evidence for a role of CDC50A-interacting P4-ATPases in the attenuation of TLR4-mediated signaling.

### CDC50A is required for LPS-induced endocytosis of TLR4 in human macrophages

**Fig. 4:**
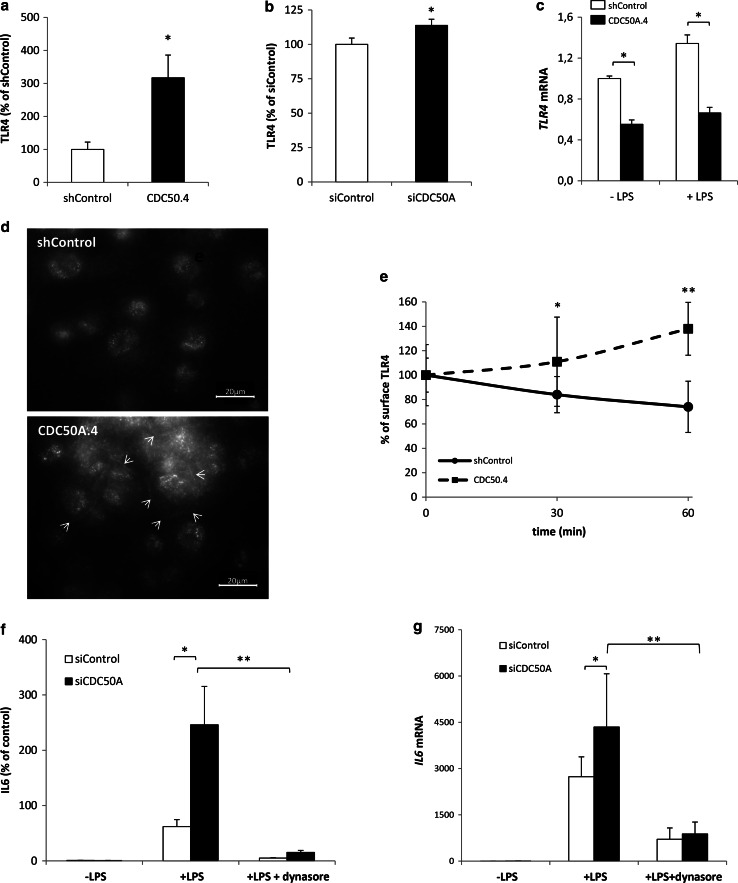
CDC50A-depleted THP-1 macrophages are impaired in LPS-induced endocytosis of TLR4. **a** TLR4 surface expression in shControl and CDC50A-depleted THP-1 macrophages was determined by flow cytometry. Data are expressed as MFI ± standard deviation of triplicate wells. Statistical significance was tested by a Student’s *t* test, *p < 0.02. **b** TLR4 surface expression in siControl and siCDC50A MDMs was determined by flow cytometry. Data are expressed as MFI ± standard deviation of triplicate wells. Statistical significance was tested by a Student’s *t* test, *p < 0.05. **c** *TLR4* mRNA levels in shControl and CDC50A-depleted THP-1 cells 3 h post-LPS administration. Statistical significance was tested by a Student’s *t* test, *p < 0.00005. **d** Immunofluorescent detection of TLR4 in shControl and CDC50A.4 cells. *Arrows* indicate TLR4 staining at the apex of the cells. **e** TLR4 surface expression in shControl and CDC50A-depleted THP-1 macrophages after stimulation with 100 ng/ml LPS. Cells were analyzed by flow cytometry. Data shown are MFIs ± standard deviation at different time points of triplicate wells. Statistical significance was determined between shControl and CDC50A.4 cells and tested by a Student’s *t* test, *p < 0.05, **p < 0.002. **f** IL6 secretion in siControl and siCDC50A MDMs (72 h post-transfection) that were stimulated for 4 h with 100 ng/ml LPS with/without 80 µM dynasore. Dynasore-treated cells were pre-incubated for 30 min with 80 µM dynasore. Data shown are means ± SEM of *n* = 8 of two different isolations. Statistical significance was tested by one-way ANOVA with Bonferroni’s correction for multiple testing, *p < 0.005; **p < 0.00005. **g** *IL6* mRNA levels in siControl and siCDC50A MDMs (72 h post-transfection) that were stimulated for 4 h with 100 ng/ml LPS with/without 80 µM dynasore. Data shown are means ± standard deviation (*n* = 8, 2 different isolations). mRNA expression in siControl cells without LPS was set as 1. Statistical significance was tested one-way ANOVA with Bonferroni’s correction for multiple testing, *p < 0.005; **p < 0.0005. Data shown in this figure are representative of three to four independent experiments with similar results

The increased and sustained activation of TLR4-dependent signaling in CDC50A-depleted macrophages led us to investigate the surface expression of TLR4 by flow cytometry. In unstimulated CDC50A-depleted THP-1 and human primary macrophages, we observed a significant 3- and 1.2-fold increase, respectively, in surface expression of TLR4 compared to controls (Fig. 4a, b), whereas *TLR4* mRNA levels were ~2 fold decreased only in THP-1 macrophages (Fig. 4c and not shown). The elevated plasma membrane localization of TLR4 was visualized by immunofluorescent staining of TLR4, which outlined the apex of CDC50A.4 cells but not that of shControl cells (Fig. 4d). Previous work has shown that TLR4 is endocytosed upon LPS stimulation, a process that is dependent on CD14 expression [4, 5, 9]. To investigate whether CDC50A was involved in TLR4 endocytosis, we stimulated CDC50A.4 macrophages with LPS and analyzed TLR4 surface expression after 30 and 60 min. While in shControl cells TLR4 surface expression was reduced by 16 % after 30 min and 26 % after 60 min, TLR4 surface expression was not reduced but increased by 38 % after 60 min in CDC50A.4 macrophages (Fig. 4e). Like TLR4, surface expression of CD14 was twofold increased in CDC50A.4 macrophages, which was mirrored by an increase at the mRNA level, however, no loss of CD14 surface expression was observed in either shControl or CDC50A.4 macrophages after LPS challenge (supplementary figure 2). These data indicate that CDC50A is essential for LPS-induced endocytosis of TLR4 in macrophages. To verify that the hyperinflammatory reaction of CDC50A-depleted cells was due to impaired TLR4 endocytosis, we chemically blocked TLR4 internalization using the GTPase dynamin inhibitor dynasore [4, 7]. We measured IL6 excretion in CDC50A-depleted human MDMs that were challenged for 4 h with LPS in the absence and presence of 80 µM dynasore. CDC50A-depleted cells displayed strongly elevated IL6 excretion when challenged with LPS alone, however, unexpectedly, dynasore treatment almost completely abolished this effect (Fig. 4f). These observations were mirrored by *IL6* mRNA expression levels (Fig. 4g). Collectively these data suggest that, apart from a role in LPS-induced internalization of TLR4, CDC50A-associated P4-ATPases may play a role in the signaling cascade downstream of TLR4.

### ATP8B1 and ATP11A-depleted THP-1 macrophages show LPS-induced hypersecretion of pro-inflammatory cytokines

**Fig. 5:**
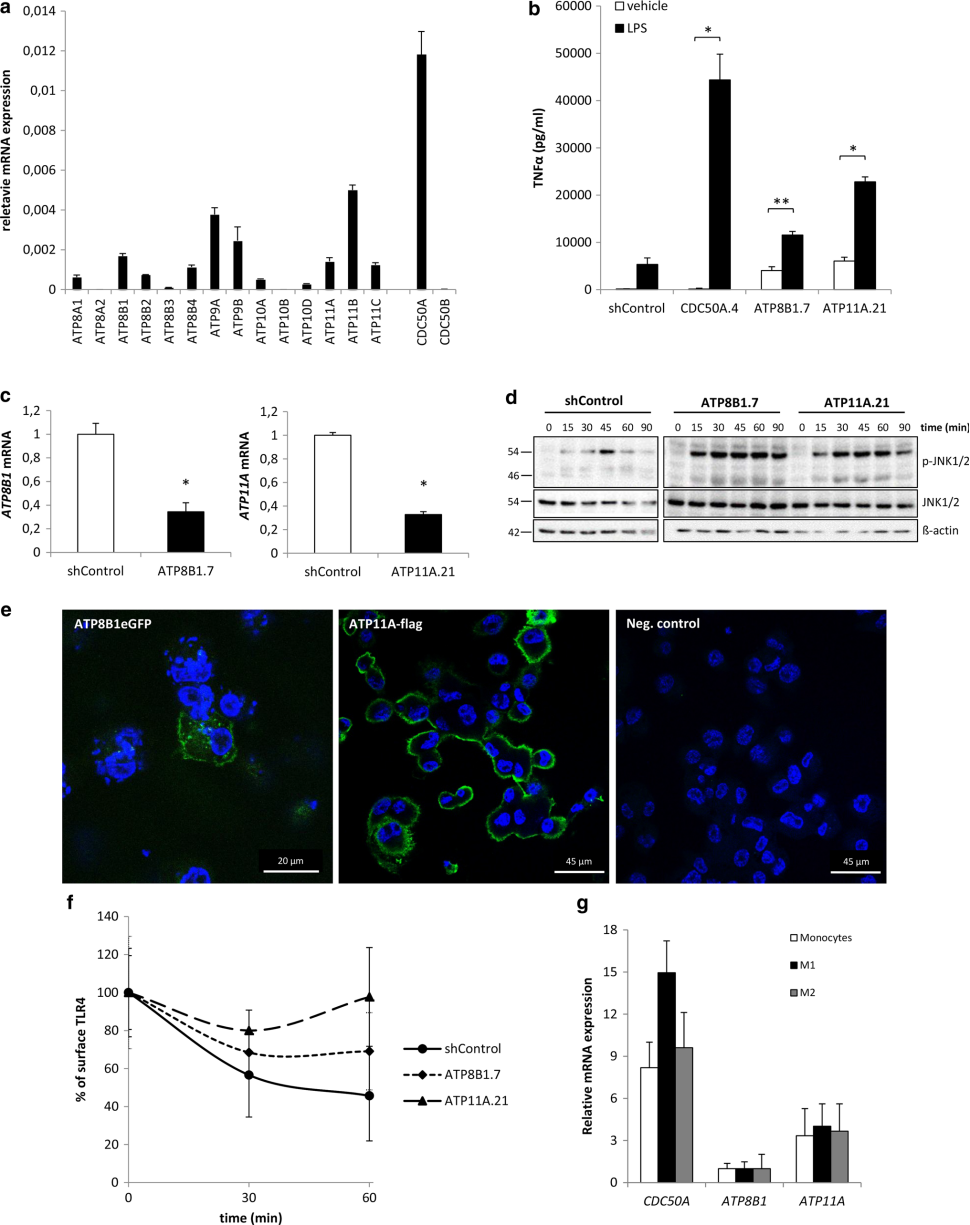
ATP8B1 and ATP11A display elevated LPS-induced TLR4-mediated signaling and reduced TLR4 internalization. **a** *P4*-*ATPases*, *CDC50A* and *CDC50B* mRNA levels in THP-1 macrophages. mRNA data shown are mean ± standard deviation of triplicate wells. **b** TNFα secretion in shControl, CDC50A-, ATP8B1- and ATP11A-depleted THP-1 macrophages 4 h after 100 ng/ml LPS administration. Data shown are mean ± standard deviation of triplicate wells. Statistical significance was tested by one-way ANOVA with Bonferroni’s correction for multiple testing, *p < 0.00001, **p = 0.05. **c** *ATP8B1* and *ATP11A* mRNA levels in shControl and ATP8B1- and-ATP11A depleted THP-1 macrophages. Data shown are mean ± standard deviation of triplicate wells. Statistical significance was tested by a Student’s *t* test, *p < 0.001. **d** Immunoblot analysis of activated JNK1/2 in shControl, ATP8B1- and ATP11A-depleted THP-1 macrophages. Cells were incubated with 100 ng/ml LPS for the time points indicated. β-Actin is included as a loading control. **e** Confocal microscopical detection of GFP-tagged ATP8B1 (ATP8B1eGFP) and flag-tagged ATP11A (ATP11A-flag) in THP-1 macrophages. Both ATP8B1eGFP and ATP11A-flag are in green and predominantly localize to the plasma membrane. Nuclear DAPI staining in *blue*. **f** TLR4 surface expression in shControl, ATP8B1- and ATP11A-depleted THP-1 macrophages after stimulation with 100 ng/ml LPS. Cells were analyzed and data expressed as described in Fig. 4a. No statistical differences by one-way ANOVA. **g** *CDC50A*, *ATP8B1* and *ATP11A* mRNA levels in primary human monocytes and M1- and M2-primed macrophages. Data shown are mean ± standard deviation of quadruple wells. *ATP8B1* expression in the three subsets was set as 1. Data shown in this figure are representative of two to three independent experiments with similar results

We analyzed which member(s) of the P4-ATPase family serve(s) as the active α-subunit for obligate heterodimerization with CDC50A. To investigate this, we depleted THP-1 cells from all individual P4-ATPases. THP-1 cells expressed 11 P4-ATPases (Fig. 5a), nine of which have been shown to heterodimerize with CDC50A [28–30]. Several P4-ATPase-depleted THP-1 cells displayed poor growth or no survival, still, seven different knockdown lines could be analyzed for the inflammatory reaction to LPS. LPS-induced TNFα excretion was ~2- and ~3-fold elevated only in ATP8B1-depleted cells (ATP8B1.7) and ATP11A-depleted cells (ATP11A.21), respectively, when compared to LPS-induced shControl macrophages (Fig. 5b). Already without LPS stimulation, TNFα output was elevated in both P4-ATPase depleted cells. The enhanced TNFα output was associated with a ~70 % reduction in *ATP8B1* and *ATP11A* mRNA expression (Fig. 5c). Similar to CDC50A.4 macrophages, ATP8B1.7 and ATP11A.21 macrophages showed an increased and more sustained JNK1/2 phosphorylation already 15 min after LPS challenge (Fig. 5d). Immunolocalization of ectopically expressed ATP8B1eGFP and ATP11A-Flag showed predominant plasma membrane localization of both proteins (Fig. 5e). Finally, we assessed TLR4 internalization 30 and 60 min after LPS administration. While after 60 min TLR4 surface expression was reduced by ~50 % in shControl cells, this was ~30 % in ATP8B1.7 cells, whereas in ATP11A.21 cells no reduction in TLR4 surface expression was observed (Fig. 5f). Both *ATP8B1* and *ATP11A* were expressed in different subsets of MDMs (Fig. 5g). These data suggest that ATP8B1 and/or ATP11A are involved in the LPS-induced internalization of TLR4 upon an LPS insult in human macrophages.

### CDC50A is involved in endotoxin tolerance in THP-1 macrophages

**Fig. 6:**
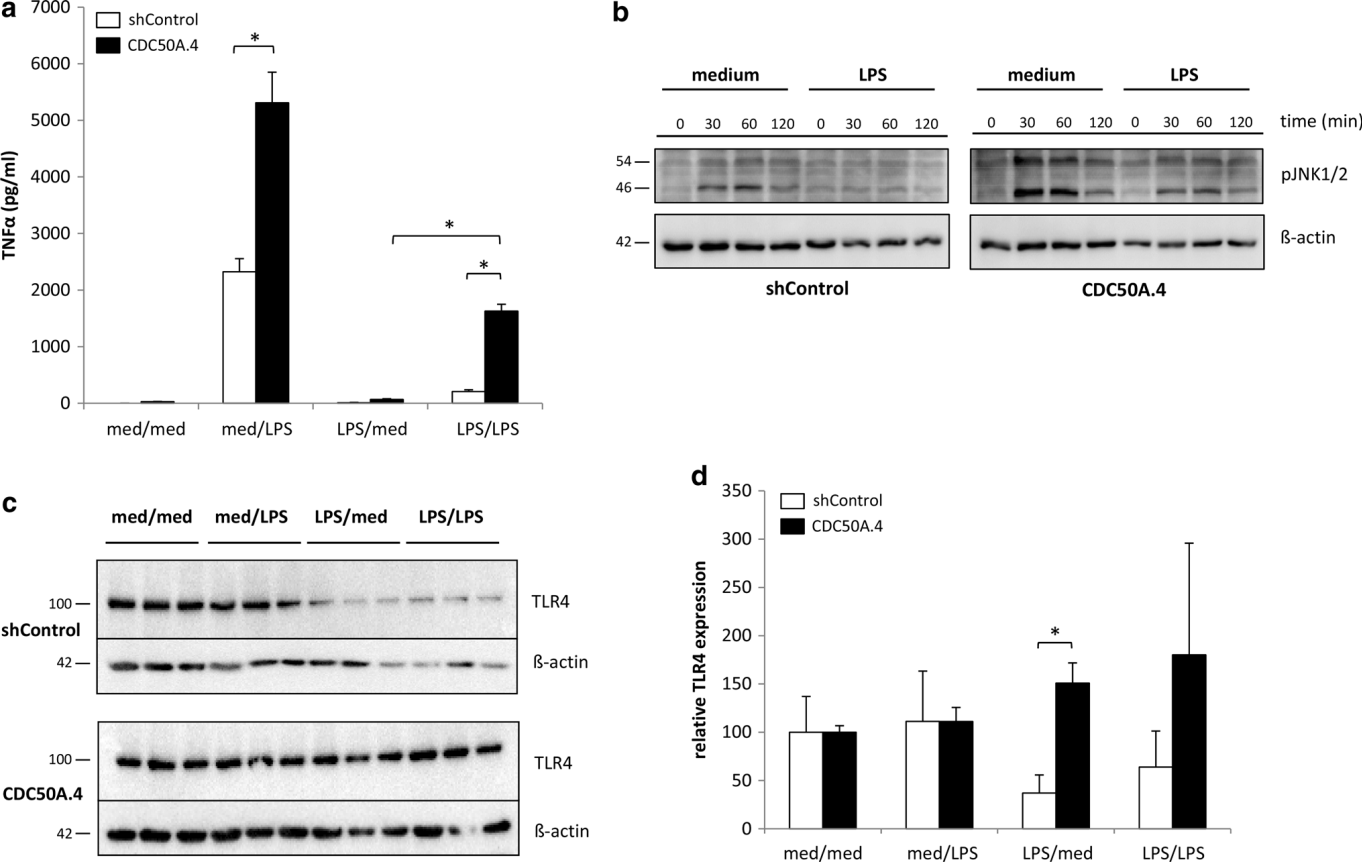
CDC50A-depleted THP-1 macrophages display a partial loss of endotoxin tolerance. **a** TNFα secretion in LPS-tolerized and non-tolerized shControl and CDC50A-depleted THP-1 macrophages. Cells were incubated with or without 100 ng/ml LPS for 18 h, washed and re-incubated with or without 100 ng/ml LPS for 6 h after which TNFα secretion was measured. Data shown are mean ± standard deviation of triplicate wells. Statistical significance was tested by one-way ANOVA with Bonferroni’s correction for multiple testing, *p < 0.00005, *med* medium. **b** LPS induced phosphorylation of JNK1/2 in tolerized and non-tolerized shControl and CDC50A-depleted THP-1 macrophages. Cells were incubated with 100 ng/ml LPS for the time points indicated and cell lysates were analysed for p-JNK1/2 expression by immunoblotting. β-Actin is included as a loading control. **c** TLR4 expression in LPS-tolerized and non-tolerized shControl and CDC50A-depleted THP-1 macrophages. Cell lysates were harvested from cells treated as described in Fig. 3a and were analyzed for TLR4 expression by immunoblotting. β-Actin is included as a loading control. **d** Quantification of total TLR4 expression in tolerized and non-tolerized shControl and CDC50A-depleted THP-1 macrophages as represented in Fig. 3c. Protein levels were quantified by densitometric analysis, normalized to β-actin, and expressed as mean percentage ± standard deviation of protein present in the med/med group (set to 100 %). Statistical significance was tested by a Student’s *t* test, *p < 0.005. Data shown in Fig. 3 are representative of two independent experiments with similar results

Because CDC50A-depleted macrophages displayed a hyper-inflammatory response to LPS, we investigated whether CDC50A could play a role in endotoxin tolerance. CDC50A-depleted THP-1 macrophages were stimulated for 18 h with 100 ng/ml LPS, washed and re-stimulated with 100 ng/ml LPS for 6 h and TNFα output and TLR4 expression were quantified. TNFα levels in the culture medium of LPS-stimulated, tolerized CDC50A.4 macrophages were ~8 times higher compared to those in LPS-stimulated tolerized shControl cells, indicating a problem with tolerization in CDC50A.4 cells (Fig. 6a). Endotoxin tolerance has been reported to be associated with reduced LPS-induced TLR4-mediated MAPK signaling in mouse macrophages [40], but also with prolonged down-regulation of TLR4 in murine and THP-1 macrophages [41, 42]. We, therefore, assessed the activation of JNK1/2 after restimulation of LPS-tolerized THP-1 cells. ShControl and CDC50A.4 cells were pretreated for 24 h with medium or 100 ng/ml LPS after which the cells were washed and restimulated with 100 ng/ml LPS for the indicated time periods. In LPS-pretreated shControl cells, we could not detect any increase in the phosphorylation status of JNK1/2 up to 120 min of LPS restimulation, which indicates that these cells were tolerant to LPS (Fig. 6b). In LPS pretreated CDC50A.4 cells, however, phosphorylated JNK1/2 was observed already 30 min after LPS restimulation indicating loss of endotoxin tolerization in these cells. Total protein levels of TLR4 were greatly reduced in tolerized shControl cells, however, in CDC50A.4 cells TLR4 expression was unaltered (Fig. 6c, quantified in Fig. 6d). These data suggest that CDC50A.4 macrophages display impaired endotoxin tolerance, which is possibly caused by sustained TLR4-mediated signaling.

## Discussion

Here we report in human THP-1 and human primary monocyte-derived macrophages that CDC50A-interacting P4-ATPase are essential to attenuate endotoxin-induced TLR4-mediated signaling possibly by facilitating the endocytic retrieval of TLR4. Endocytosis of TLR4 is not only crucial to prevent severe chronic inflammatory conditions such as sepsis, but also to guarantee a sustained immune response to suppress an ongoing infection. It is well documented that activation of TLR4 sequentially induces two signaling pathways leading to an early and a late inflammatory response [1]. The early response, activated by ligand binding and dimerization of TLR4, initiates signaling via NF-κB and the MAP kinase (MAPK) pathway and leads to the production of pro-inflammatory cytokines. Our data show that depletion of CDC50A, which is an essential subunit of P4-ATPase phospholipid flippase complexes that localize to (amongst others) the plasma membrane [27–30], results in a hyperactivation of the early response pathway as evidenced by elevated TNFα, IL-1β and IL6 release, enhanced MAPK signaling and sustained NF-κB activation. The late response is activated upon ligand-induced TLR4 endocytosis and leads to a type I interferon response [4, 5, 7, 43]. We show here that in CDC50A-depleted cells the LPS-induced internalization of TLR4 is impaired and that the induction of the interferon response in these cells is reduced; The lack of complete ablation of the interferon response in our study can be explained by residual expression of CDC50A in the knockdown cells and/or the timing of LPS treatment (3 h in our study).

Collectively, our data suggest that LPS-induced hyperactivation of Myd88-dependent signaling in CDC50A-depleted cells is caused by impaired endocytic retrieval of TLR4 leading to sustained TLR4 signaling. This hypothesis is supported by previous studies in CD14-deficient mouse bone marrow-derived macrophages (BMDM), CD14 being crucial for LPS-induced endocytic retrieval of TLR4 [5, 9]. In CD14-deficient BMDMs, TNFα excretion was almost completely abolished at low dose LPS (10 ng/ml), however, at higher LPS doses (≥100 ng/ml) TNFα excretion was increased compared to wild type cells, an observation that remained unexplained [5, 9], but that may be caused by sustained TLR4 signaling. In our study we also applied 100 ng/ml LPS, which resulted in increased cytokine excretion by CDC50A-depleted cells. We can, however, not exclude that the phenotype in CDC50A-depleted macrophages is caused by two independent processes, i.e., impaired TLR4 internalization and sustained activation of the signaling cascade downstream TLR4. This is underscored by the unexpected observation that inhibition of LPS-induced TLR4 internalization by the dynamin inhibitor dynasore completely abrogated IL6 excretion and mRNA expression in CDC50A-depleted macrophages. This observation suggests that the hyper-responsiveness of CDC50A-depleted cells to LPS was not caused by impaired TLR4 internalization. However, the specificity of dynasore, i.e., a selective inhibitor of the dynamin GTPase activity to inhibit clathrin-mediated endocytosis, has recently being disputed [44]. Multiple dynamin-independent effects of dynasore have been described, including disruption of lipid rafts i.e., detergent-resistant membrane domains that serve as signaling platforms [45, 46]. Previous studies have shown that following LPS stimulation, TLR4 and CD14 are mobilized to lipid rafts, an event crucial for TLR4 dimerization and subsequent recruitment and assembly of adaptor proteins; The use of lipid raft-disrupting compounds abrogates the LPS-induced TLR4 signaling [47, 48]. Thus, dynasore treatment of macrophages may not only inhibit clathrin-mediated endocytosis of TLR4 but also the lipid raft-associated TLR4 signaling cascade, explaining the lack of inflammatory reaction in dynasore-treated CDC50A-depleted cells. Still, we cannot exclude the possibility that, apart from blocking TLR4 endocytosis, CDC50A depletion may interfere with Myd88-dependent signaling downstream of TLR4.

We showed that CDC50A-depleted macrophages were impaired in the LPS-induced internalization of TLR4, however, in the TLR4 endocytosis assay TLR4 surface expression was increased 30 and 60 min after LPS challenge compared to unchallenged cells. Although we have no clear explanation for this observation it may be that LPS challenge mobilizes an existing pool of TLR4 to the plasma membrane that subsequently cannot be internalized leading to an increase of TLR4 surface expression post-LPS challenge. Furthermore, and despite increased TLR4 surface expression, *TLR4* mRNA levels were reduced in CDC50A-depleted cells, which may be due to the sustained activation of the Myd88-dependent signaling pathway leading to a compensatory down-regulation of *TLR4* transcription. Previous studies showed that LPS-induced internalization of TLR4 associates with endocytosis of CD14 [5, 49], although Rajaiah et al. [9] recently reported CD14-independent TLR4 endocytosis. We did not observe any reduction in CD14 surface expression in LPS-challenged control macrophages. Still, CD14 surface expression in CDC50A-depleted cells was strongly increased compared to control cells. An explanation for this observation may be that, due to reduced TLR4 turn-over, the cell responds by elevating *CD14* transcription with consequent elevation of CD14 surface levels. Indeed, we found that *CD14* mRNA levels were increased in CDC50-depleted cells.

CDC50A-depleted cells also displayed a partial loss of endotoxin tolerance. Endotoxin tolerance is a condition of reduced responsiveness to LPS upon a secondary LPS challenge with concomitant repression of proinflammatory cytokine production [50]. Tolerization, which is crucial for dampening a recurrent inflammatory insult and protects the host to fatal infection (i.e, during sceptic shock), is associated with repression of TLR4-mediated signaling and reduced TLR4 surface expression [40–42, 50]. Reduced endotoxin tolerance in CDC50A-depleted cells coincided with enhanced JNK1/2 activation and TNFα excretion upon restimulation with LPS. Furthermore, whereas in control cells total TLR4 protein levels were reduced after overnight LPS challenge, TLR4 protein levels were unaffected in CDC50A-depleted cells. Apparently, sustained TLR4 surface expression accounts for the reduced tolerized state in CDC50A-depleted cells.

CDC50A-depleted cells were phenocopied by ATP8B1- and ATP11A-depleted cells with regard to enhanced LPS-induced JNK1/2 activation, TNFα output and impaired LPS-induced internalization of TLR4. This underscores the need for obligate heterodimerization of a member of the P4-ATPase family of phospholipid flippases with CDC50A to form an active phospholipid flippase complex. These phenotypes were, however, not as dramatic as in CDC50A-depleted cells, which can be best explained by residual activity of these P4-ATPases or activity of other, redundant P4-ATPases.

How are P4-ATPases involved in the endocytic retrieval of TLR4? Although CDC50A was detected in intracellular vesicles, both ATP8B1 and ATP11A predominantly localized to the plasma membrane of THP-1 cells, which renders a role in the endocytic retrieval, recycling and/or TGN-to plasma membrane delivery likely. One of the preferred canonical substrates for ATP8B1 and ATP11A is phosphatidylserine (PS) [12, 27, 51], although recently phosphatidylcholine (PC) has been proposed to be a substrate for ATP8B1 as well [51]. PS has been implicated to drive endocytic processes in human and yeast cells [52–54]; for instance, Farge et al. have shown that the formation of endocytic vesicles was enhanced when the cytosolic surface area was expanded upon incubation of the cells with PS and PE analogs, a process that depended on a plasma membrane flippase activity [52]. P4-ATPases can catalyze a local concentration of phospholipids and the consequential vesiculation according to the *bilayer couple hypothesis* [55]. This hypothesis proposes that if one leaflet of a bilayer expands through a local increase in phospholipids, the coupled leaflet follows, which leads to bending of the bilayer. The resulting membrane curvature facilitates binding of curvature-stabilizing proteins and/or proteins of the vesicle-generating machinery. For instance, F-BAR domain-containing proteins sense and stabilize shallow membrane curvatures in the early steps of membrane invagination and contain positively charged membrane binding domains that interact with PS [56–58]. In addition, the negative charge of the serine head group in PS is important for recruiting positively charged proteins of the vesicle-generating machinery to initiation sites within the endocytic pathway [59]. Such a mechanism has recently been confirmed by Xu et al. [60] who showed that localized PS flipping by the *S. cerevisiae* P4-ATPase Drs2p creates a negatively charged, curved membrane structure that recruits the ADP-ribosylation factor GTPase activating protein (ARF-GAP) Gcs1, a major regulator of the vesicle-generating machinery [61]. In addition, Lui et al. previously demonstrated an essential role for the PS flippase Drs2p in the formation of clathrin-coated vesicles at the TGN [62]. Very recently Bruurs et al. have shown that ATP8B1 is essential for clustering of Cdc42, which can interact with PS [63], during the establishment of the apical membrane of enterocytes [64]. Based on these observations, we hypothesize that ATP8B1 and/or ATP11A mediate local clustering of PS or other phospholipids at the plasma membrane to induce a curvature which facilitates the binding of proteins of the vesicle-generating machinery, including clathrin. This is the initiating event in the biogenesis of clathrin-coated pits via which LPS-ligated TLR4 is endocytosed. Indeed, Husebye et al. previously reported that endocytosis of TLR4 relies on clathrin [4].

Here we report an essential role for CDC50A-associated phospholipid flippases in the innate immune response. Although it remains to be determined how these proteins are involved in the inflammatory reaction and what the relative contribution is to this process, we have identified two potential candidates i.e., ATP8B1 and ATP11A, both of which are expressed in human macrophages. Deficiency of ATP8B1 or ATP11A is associated with chronic inflammatory conditions in humans. For instance, patients with ATP8B1 deficiency present with severe chronic liver disease but are also susceptible to pneumonia [65, 66]. Previously, it was proposed that pulmonary infection in ATP8B1 deficiency causes pulmonary cardiolipin levels to rise leading to impaired lung (surfactant) function, leading to pulmonary inflammation, a hypothesis that was debated by us [67, 68]. Based on our present work, an alternative explanation can be that patients have problems attenuating the TLR4-mediated inflammatory reaction in pulmonary macrophages. Similarly, in a recent genome-wide association study ATP11A was associated with fibrotic idiopathic interstitial pneumonias, a group of pulmonary disorders associated with inflammation and fibrosis [69]. Whether P4-ATPase genes are novel risk genes for inflammatory disease remains to be established.

### Electronic supplementary material

Below is the link to the electronic supplementary material.

Supplementary material Table 1 (PDF 33 kb)

**Supplementary Figure 1** (**A**) CD11B surface expression in shControl and CDC50A-depleted THP-1 macrophages was determined by flow cytometry. Data are expressed as mean fluorescence intensity (MFI) ± standard deviation of triplicate wells. No statistical differences by a Student’s t-test. (**B**) TNFα excretion and *CDC50A* mRNA expression in CDC50A-depleted THP-1 cells 4 h post LPS (100 ng/ml). Statistical significance was tested by one-way ANOVA with Bonferroni’s correction for multiple testing; *p< 0,05; **p < 0,0005 (**C**) *CD80* and *CD200R* mRNA expression in M0, M1 and M2 human monocyte-derived macrophages. (TIFF 1893 kb)

**Supplementary Figure 2** (**A**) CD14 surface expression in shControl and CDC50A-depleted THP-1 macrophages was determined by flow cytometry. Data shown are MFIs ± standard deviation of triplicate wells. Statistical significance was tested by a Student’s t-test, *p<0.00005. (**B**) *CD14* mRNA levels in shControl and CDC50A-depleted THP-1 cells 3 hours post-LPS administration. Statistical significance was tested by a Student’s t-test, *p<0.002, **p<0.008. (**C**) CD14 surface expression in shControl and CDC50A-depleted THP-1 macrophages after stimulation with 100 ng/ml LPS. Cells were analyzed and data expressed as described in figure 4**A** (TIFF 1436 kb)
