# Supplementary material for: Phospholipid flippases attenuate LPS-induced TLR4 signaling by mediating endocytic retrieval of Toll-like receptor 4
Source: Cell Mol Life Sci. 2016 Sep 14;74(4):715–30. doi: 10.1007/s00018-016-2360-5 (PMC5272906; doi:10.1007/s00018-016-2360-5)
Supplement: Supplementary file 1 — Supplementary material Table 1 (PDF 33 kb) [file 18_2016_2360_MOESM1_ESM.pdf]

TARGET	FORWARD (5’-3’)	REVERSE (5’-3’)
**HUMAN**		
ATP8A1	TGCAGCAAATACCTGATGTGT	AGTGGACAATTTCCCAAGCAC
ATP8A2	AAGATTTTAAGCGACACAAGGCA	GACGACCTTCACAATGTCTCC
ATP8B1	GTCTTGGACAGAGTCACTTC	CGTCTTATCAGAGAAGATATAAT
ATP8B2	TCTCTCCGACACGGTCCGCT	AGCTCCCCGAAGCCCTCCTG
ATP8B3	CGCCCTGAACCTCCCTTTTC	GACCTGAGCCTTCCTGAGTC
ATP8B4	AAAAGTCCGCCATGCACTATC	ACAACAATCCCATCAAACCCTG
ATP9A	ATAGGCCGTGCGTTCGCTGG	AGGCGCAGAGCCACTTCCCA
ATP9B	GGGGCCCTGGTGCTCTTCGA	CGGCCACCACCATCAGCCAG
ATP10A	CTCCGACCACAAGATCAACCA	CGAAGACGCAGAAAGTCTCC
ATP10B	ATGCATTGCCAAGAAGGTTG	TGCTGTGCAGTTTCCATGAG
ATP10D	CCATGAAGAAAATGCCAGGA	TGCCATATTGCTGAGGGAAC
ATP11A	AACGCAGGAGGGACATTCTG	CCTGGACGGCTCTTCTCTC
ATP11B	TGGCCATTTTTGGGCTCCCAGA	TGTAGGGTGGAGGTGTCGGTCA
ATP11C	CAATGCCATGCTACAGTTGGG	GAATCGGGTATCCAAGGCAAG
CDC50A (TMEM30A)	AGCTGGCCGATACTCTTTGA	GAGATGGATCCAACAGCGAT
CDC50B (TMEM30B)	GCCACAAGCTCCTCATCTTC	CTGGTCCTGGTAGCGAATGT
TNFα	CTGCTGCACTTTGGAGTGAT	AGATGATCTGACTGCCTGGG
IL1ß	GAAGCTGATGGCCCTAAACA	AAGCCCTTGCTGTAGTGGTG
IL6	CCAGAGCTGTGCAGATGAGT	CTGCAGCCACTGGTTCTGT
INFß	ATGACCAACAAGTGTCTCCTCC	GGAATCCAAGCAAGTTGTAGCTC
RANTES (CCL5)	CCTGCTGCTTTGCCTACATT	ACACACTTGGCGGTTCTTC
SOCS1	CTACCTGAGCTCCTTCCCCT	CACATGGTTCCAGGCAAGTA
TLR4	AGGGAGACACAGATGGCTGGGA	AGGAGCATTGCCCAACAGGAAACA
CD14	TACTGGTAGGCGCCCTGCGT	AGCCAAGAACGCCCTGTCGC
RPLP0	TCGACAATGGCAGCATCTAC	ATCCGTCTCCACAGACAAGG
HPRT	CATTATGCTGAGGATTTGGAAAGG	CTTGAGCACACAGAGGGCTACA
ACTB	AATGTGGCCGAGGACTTTGA	TGGCTTTTAGGATGGCAAGG
GAPDH	GAGTCAACGGATTTGGTCGT	TTGATTTTGGAGGGATCTCG
CD80	CTGCCTGACCTACTGCTTTG	GGCGTACACTTTCCCTTCTC
CD200R	GAGCAATGGCACAGTGACTGTT	GTGGCAGGTCACGGTAGACA
**MOUSE**		
TNFα	TGGAACTGGCAGAAGAGGCACT	CCATAGAACTGATGAGAGGGAGGC
IL1ß	CTCGTGCTGTCGGACCCAT	TGCCGTCTTTCATTACACAGGA
RANTES (CCL5)	CTGCTGCTTTGCCTACCTCT	CACTTCTTCTCTGGGTTGGC
RPLP0	GGACCCGAGAAGACCTCCTT	GCACATCACTCAGAATTTCAATGG

Supplemental table 1. Oligonucleotide sequences used for quantitative RT-PCR experiments.
