# Supplementary figures and images for: Phospholipid flippases attenuate LPS-induced TLR4 signaling by mediating endocytic retrieval of Toll-like receptor 4
Source: Cell Mol Life Sci. 2016 Sep 14;74(4):715–30. doi: 10.1007/s00018-016-2360-5 (PMC5272906; doi:10.1007/s00018-016-2360-5)

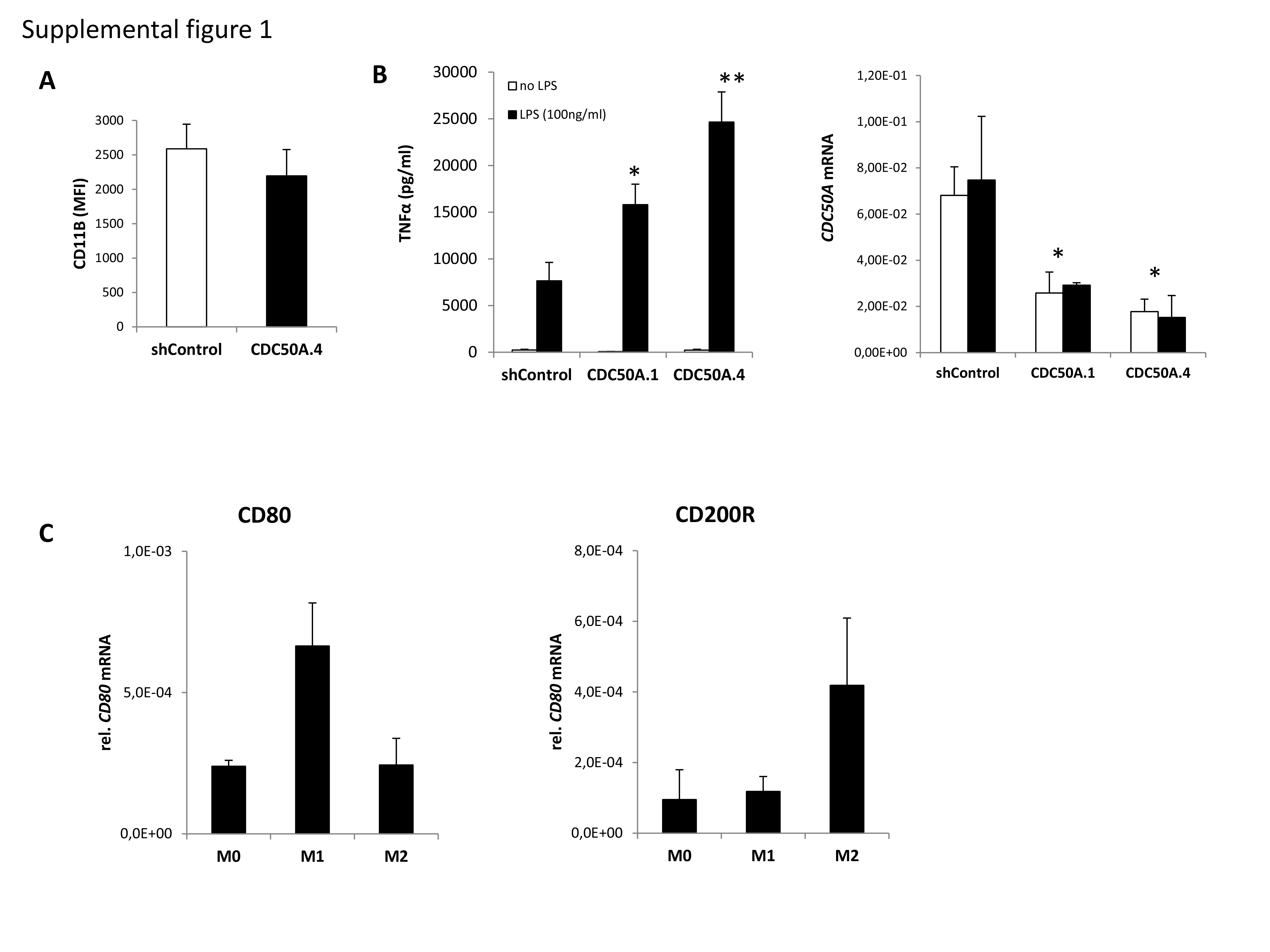

Supplement: Supplementary file 2 — Supplementary Figure 1 (A) CD11B surface expression in shControl and CDC50A-depleted THP-1 macrophages was determined by flow cytometry. Data are expressed as mean fluorescence intensity (MFI) ± standard deviation of triplicate wells. No statistical differences by a Student’s t-test. (B) TNFα excretion and CDC50A mRNA expression in CDC50A-depleted THP-1 cells 4 h post LPS (100 ng/ml). Statistical significance was tested by one-way ANOVA with Bonferroni’s correction for multiple testing; *p< 0,05; **p < 0,0005 (C) CD80 and CD200R mRNA expression in M0, M1 and M2 human monocyte-derived macrophages. (TIFF 1893 kb) [file 18_2016_2360_MOESM2_ESM.tif]

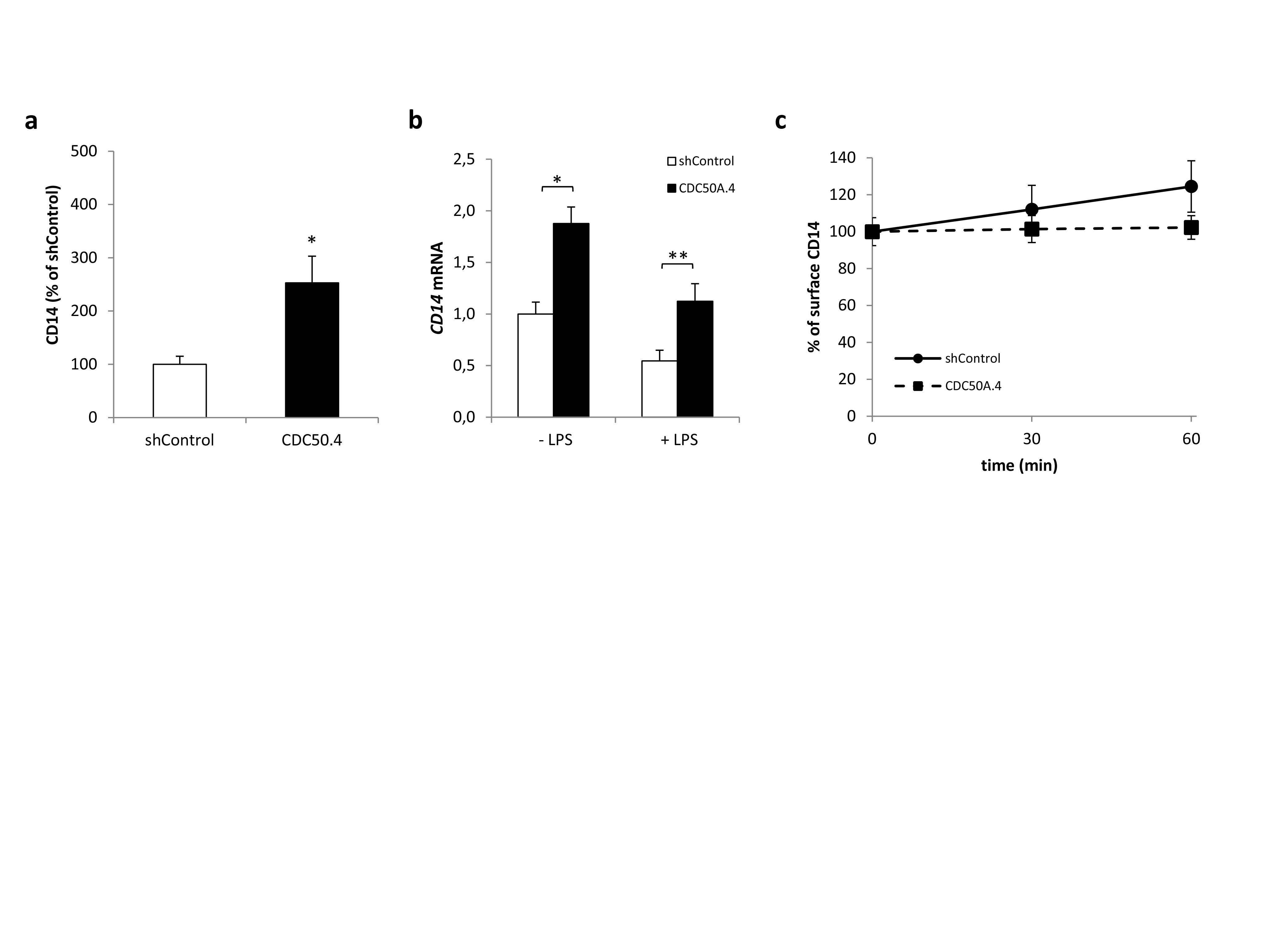

Supplement: Supplementary file 3 — Supplementary Figure 2 (A) CD14 surface expression in shControl and CDC50A-depleted THP-1 macrophages was determined by flow cytometry. Data shown are MFIs ± standard deviation of triplicate wells. Statistical significance was tested by a Student’s t-test, *p<0.00005. (B) CD14 mRNA levels in shControl and CDC50A-depleted THP-1 cells 3 hours post-LPS administration. Statistical significance was tested by a Student’s t-test, *p<0.002, **p<0.008. (C) CD14 surface expression in shControl and CDC50A-depleted THP-1 macrophages after stimulation with 100 ng/ml LPS. Cells were analyzed and data expressed as described in figure 4A (TIFF 1436 kb) [file 18_2016_2360_MOESM3_ESM.tif]
